# Collagen concentration regulates neutrophil extravasation and migration in response to infection in an endothelium dependent manner

**DOI:** 10.3389/fimmu.2024.1405364

**Published:** 2024-07-03

**Authors:** Christopher J. Calo, Tanvi Patil, Mallory Palizzi, Nicola Wheeler, Laurel E. Hind

**Affiliations:** Department of Chemical and Biological Engineering, University of Colorado Boulder, Boulder, CO, United States

**Keywords:** neutrophil, collagen, microfluidic, endothelium, infection, microphysiological system, infection-on-a-chip

## Abstract

**Introduction:**

As the body’s first line of defense against disease and infection, neutrophils must efficiently navigate to sites of inflammation; however, neutrophil dysregulation contributes to the pathogenesis of numerous diseases that leave people susceptible to infections. Many of these diseases are also associated with changes to the protein composition of the extracellular matrix. While it is known that neutrophils and endothelial cells, which play a key role in neutrophil activation, are sensitive to the mechanical and structural properties of the extracellular matrix, our understanding of how protein composition in the matrix affects the neutrophil response to infection is incomplete.

**Methods:**

To investigate the effects of extracellular matrix composition on the neutrophil response to infection, we used an infection-on-a-chip microfluidic device that replicates a portion of a blood vessel endothelium surrounded by a model extracellular matrix. Model blood vessels were fabricated by seeding human umbilical vein endothelial cells on 2, 4, or 6 mg/mL type I collagen hydrogels. Primary human neutrophils were loaded into the endothelial lumens and stimulated by adding the bacterial pathogen *Pseudomonas aeruginosa* to the surrounding matrix.

**Results:**

Collagen concentration did not affect the cell density or barrier function of the endothelial lumens. Upon infectious challenge, we found greater neutrophil extravasation into the 4 mg/mL collagen gels compared to the 6 mg/mL collagen gels. We further found that extravasated neutrophils had the highest migration speed and distance in 2mg/mL gels and that these values decreased with increasing collagen concentration. However, these phenomena were not observed in the absence of an endothelial lumen. Lastly, no differences in the percent of extravasated neutrophils producing reactive oxygen species were observed across the various collagen concentrations.

**Discussion:**

Our study suggests that neutrophil extravasation and migration in response to an infectious challenge are regulated by collagen concentration in an endothelial cell-dependent manner. The results demonstrate how the mechanical and structural aspects of the tissue microenvironment affect the neutrophil response to infection. Additionally, these findings underscore the importance of developing and using microphysiological systems for studying the regulatory factors that govern the neutrophil response.

## 1 Introduction

As the body’s first cellular line of defense against bacterial and fungal infections, neutrophils play a crucial role in innate immunity. Unfortunately, dysregulated neutrophil function is implicated in the pathogenesis of many diseases and conditions, including cancer, fibrosis, aberrant wound healing, and aging (1–7). Individuals afflicted with these conditions are also more susceptible to severe infections; therefore, recent studies have highlighted the potential of neutrophils as therapeutic targets (8–12). However, to effectively design such therapeutics, it is imperative to understand the regulatory factors that govern the neutrophil response. While the chemical factors that regulate the neutrophil response have been well studied, our understanding of how the physical environment affects the response remains incomplete. Interestingly, diseases worsened by neutrophil dysregulation are often also accompanied by changes in the protein composition of the local extracellular matrix (ECM) (13–16). These changes include increased collagen deposition as well as decreased enzymatic turnover of collagen present in the matrix. Thus, in this study we sought to determine how increasing collagen concentration affects the neutrophil response to infection.

Type I collagen is the predominant protein the ECM, providing scaffolding and mechanical stability to most tissues (17). As such, changing the concentration of collagen in the ECM alters both the structural and mechanical properties of the environment. Several studies employing hydrogel systems with tunable mechanical properties have shown that neutrophils are mechanosensitive (18–26). Specifically, it has been reported that neutrophil extravasation increases as the substrate stiffness underlying the endothelium increases (23). Additionally, in these systems, optimal neutrophil motility is dependent on both the substrate stiffness and the concentration of matrix protein coating the surface (19). In three dimensions (3D), neutrophil migration through collagen matrices increases as the cross-sectional area of the pores in the matrix increases (27). Mechanical and structural signals from the environment have also been shown to affect the antimicrobial functions of neutrophils. When seeded on substrates of increasing stiffness, neutrophils increase neutrophil extracellular trap formation (25, 28). Furthermore, neutrophil binding to type I collagen via β1 integrins on the cell surface has been demonstrated to increase reactive oxygen species production (29–31). Thus, it is possible that changes to the composition of the ECM could have a profound effect on multiple aspects of the neutrophil response.

Neutrophil-endothelial cell interactions are crucial for initiating the neutrophil response (32, 33). Like neutrophils, endothelial cells have been shown to be sensitive to the mechanical and structural properties of the substrates on which they are seeded (34–39). Therefore, it is important to understand how changes to the ECM affect the endothelium, as that could indirectly cause differential activation of neutrophils. It has been shown that the stiffness of an endothelial layer directly correlates with the stiffness of the underlying substrate (36). Endothelial stiffening has been shown to increase permeability by increasing cell-cell junction distance (40) and to alter secretion profiles of angiogenic and inflammation-related cytokines, including MCP-1, IL-4, and IL-3 (41). Furthermore, Bischel, et al. demonstrated that endothelial cells change their protein secretion profiles in differing monolayer geometries (42). These studies highlight the importance of incorporating a physiologically relevant model endothelium when investigating the effects of ECM composition on the neutrophil response. Using a physiologically relevant model also allows us to probe both direct (neutrophil-ECM interactions) and indirect (endothelial cell-ECM interactions) mechanisms by which the ECM regulates neutrophil function.

While many studies have investigated how ECM composition affects the neutrophil response, few have studied the response from a wholistic perspective in a physiologically relevant environment. This is, in part, due to a lack of tools for evaluating such effects. Many neutrophil motility studies were conducted with 2D systems; however, it has been shown that neutrophil polarity is differentially regulated in 2D versus 3D environments (43). Traditional *in vitro* models afford researchers control over the ECM but lack relevant physiological components, such as a 3D endothelial lumen or a tissue matrix through which neutrophils can migrate. In contrast, *in vivo* models provide pertinent physiological aspects of the system; yet they afford minimal, if any, control over the ECM. Therefore, recent studies have investigated tissue microenvironments with microphysiological devices that allow for control over the ECM composition while also incorporating physiologically relevant aspects of the tissue microenvironment (44–50).

In this study, we used our recently developed infection-on-a-chip microfluidic device, which replicates a portion of a blood vessel surrounded by a model ECM, to investigate the effects of collagen concentration on the neutrophil response to *Pseudomonas aeruginosa*, an opportunistic pathogen that affects immunocompromised people, such as those with cancers or fibrosis. We found greater neutrophil extravasation out of endothelial lumens seeded in 4 mg/mL collagen compared to lumens seeded in 6 mg/mL collagen, and migration of extravasated neutrophils decreased with increasing collagen concentration. However, these differences in extravasation and migration were not observed in the absence of an endothelium, suggesting an endothelial cell-dependent mechanism. Lastly, no significant differences were seen in the percentage of extravasated neutrophils producing reactive oxygen species (ROS). Together, these results underscore the importance of studying physical regulators of the neutrophil response and indicate a role for collagen concentration in regulating neutrophil extravasation and migration in an endothelial cell-dependent manner.

## 2 Materials and methods

### 2.1 Microfluidic fabrication

The microfluidic devices were fabricated as previously described (51). In brief, SU-8–100 (Y131273, Kayaku Advanced Materials, Westborough, MA) was patterned onto silicon wafers (857, University Wafer Inc., Boston, MA) using soft lithography to create a top and bottom master for the devices. Polydimethylsiloxane (PDMS, 24236–10, Electron Microscopy Sciences, Hatfield, PA) was then polymerized over the masters for 4 hours at 60 °C. Then, the PDMS layers were aligned, and a PDMS rod (0.337 mm inner diameter) was inserted between the two layers before the devices were bonded to a glass-bottom dish (P50G-1.5–30-F, MatTek Corporation, Ashland, MA) with oxygen plasma from a PE25-JW Plasma Etcher (Plasma Etch, Carson City, NV).

### 2.2 HUVEC culture

Pooled human umbilical vein endothelial cells (HUVEC, 50–305-964, Promocell GmbH C12203, Heidelerg, Germany) were cultured in Endothelial Growth Medium 2 (EGM-2, NC9525043, Lonza Walkersville CC3162, Basel, Switzerland). Media was changed every 2 days. HUVECs were seeded on cell culture-treated flasks (658175, Greiner Bio-one, Monroe, NC), passed at 80% confluency, and used through passage 7.

### 2.3 *Pseudomonas aeruginosa* culture

LB agar (22700025, Thermo Fisher Scientific, Waltham, MA) was used according to the manufacturer’s instructions to create LB plates. *Pseudomonas aeruginosa* strain K (PAK) was streaked onto LB plates and incubated at 37 °C for 16 hours. The plates were then placed into a refrigerator at 4 °C until they were used. A single colony was grown overnight in 5 mL of LB broth in a bacterial shaker at 37 °C. The next day the 1 mL of the cultured solution was diluted in 4 mL of fresh LB broth and grown in a bacteria shaker at 37 °C for 1.5 hours. 1 mL of bacterial culture was pelleted by centrifugation (17,000xg for 1 minute) and resuspended in 100 μL of EGM-2. The optical density (OD) was measured, and the bacterial solution was diluted in EGM-2 to an OD of 5 (measured at 600 nm, 1.25 x 10^6^ CFU/mL). The EGM-2 contains gentamicin, which prevents replication of the bacteria.

### 2.4 Device and collagen preparation

The microfluidic devices were UV sterilized for 15 minutes in a biosafety cabinet. Subsequently, the central chambers were incubated with a 2% polyethylenimine solution in deionized water (03880, Sigma Aldrich, St. Louis, MO) for 10 minutes at room temperature, followed by incubation with a 0.4% glutaraldehyde solution in deionized water (G6257, Sigma Aldrich, St. Louis, MO) for 30 minutes at room temperature. High concentration type-I collagen (354249, Corning, Corning, NY) was neutralized to pH 7.2 using a combination of 0.5 N NaOH (02153495.5, MP Biomedicals Inc., Pittsburgh, PA), 1X phosphate buffered saline (PBS, 10010049 Gibco, Waltham, MA), and 10X PBS (BP2944100, Fisher BioReagants, Pittsburgh, PA) with a final concentration of 2, 4, or 6 mg/mL. The collagen was then loaded into the device chambers around the PDMS rod. After allowing the collagen to polymerize overnight at 37 °C, tweezers were used to remove the PDMS rods, leaving a cylindrical void in the collagen network. HUVECs were seeded in these lumens at a concentration of 20,000 cells/μL. The devices were flipped every 5 minutes for 30 minutes before being placed on a rotator overnight at 37 °C with 5% CO_2_. The devices were cultured for two days, and media was changed twice daily. For experiments without an endothelium, the HUVEC seeding and rotating steps were skipped.

### 2.5 Rheological gel characterization

All characterization was performed using a Discovery HR20 rheometer (TA Instruments, New Castle, DE) fitted with a 20 mm diameter cone geometry, 1° cone angle, and a 23 μm gap. Collagen solutions were prepared as described above. Oscillatory time sweeps (10 rad/s, 1% strain) were performed with an initial temperature of 10 °C, which was increased to 37 °C within the first 70 seconds of each test. Stress relaxation experiments (15% strain) were conducted at 37 °C.

### 2.6 Confocal reflectance microscopy

Collagen pores were visualized via confocal reflectance as described previously (52). In brief, images were obtained with a Nikon A1R HD25 Laser Scanning Confocal Microscope along with a 405 nm laser and a Nikon 20x/0.95 (NA) water immersion objective operated by Nikon Elements software.

### 2.7 Pore size analysis

Images were loaded into Matlab (MathWorks, 2023b) and preprocessed to remove background noise using a Wiener filter (0.5 x 0.5 μm neighborhood) and a TopHat filter (1.0 μm strel disk diameter). Then, the images were converted to binaries, such that fibers had pixel values of 0 and pores had pixel values of 1. Binaries were eroded with progressively larger disk sizes until a threshold of 50% erosion was crossed. Clusters of pixels with the same values were grouped as objects and the regionprops function in Matlab was used to get the areas and minor axis lengths of each pore.

### 2.8 Stained endothelial lumens

After 2 days of culture in the microfluidic devices, HUVEC lumens were incubated with prewarmed 4% paraformaldehyde (PFA, AAJ19943K2, Thermo Scientific, Waltham, MA) in phosphate buffered saline (PBS, B2944–100, Thermo Scientific, Waltham, MA) for 30 minutes at 37 °C. The PFA was removed with 3 washes of PBS. Next, PBST (PBS with 0.1% Tween 20) was added to the lumens and left to incubate at room temperature for 10 minutes. Lumens were then incubated with 0.005 mg/mL Hoescht (23491–45-4, Sigma Aldrich, St. Louis, MO), phalloidin (1:200, ab176757, abcam, Cambridge, United Kingdom), and anti-VE-cadherin (1:120, 130–123-688, Miltenyi Biotec, Bergisch Gladbach, Germany) in PBST overnight at 4 °C. The next morning the lumens were washed 3 times with PBS to remove any unconjugated staining molecules. Single timepoint Z-stacks were taken with 2 μm steps along the Z-axis with a Nikon A1R HD25 Laser Scanning Confocal Microscope along a Nikon 20x/0.95 (NA) water immersion objective operated by Nikon Elements software.

For visualizing ICAM-1 expression the above protocol was followed except 3 μL of PAK in EGM-2 was added to the top port of each device and allowed to incubate for 2 hours before fixing with 4% PFA. The devices were stained with 0.005 mg/mL Hoescht (23491–45-4, Sigma Aldrich, St. Louis, MO), phalloidin (1:200, ab176757, abcam, Cambridge, United Kingdom), and anti-ICAM-1 (1:200, BBA20, R&D systems, Minneapolis, Minnesota).

### 2.9 Nuclei count

HUVECs in the microfluidic devices were stained and imaged as described above with Hoechst, anti-VE-cadherin, and phalloidin. A maximum intensity projection along the Z-axis was created for the bottom half of each lumen. The number of nuclei in a 450 x 250 μm region of interest placed at the center of each lumen were counted.

### 2.10 ICAM-1 intensity

HUVECs in the microfluidic devices were stained and imaged as described above with Hoechst, anti-ICAM-1, and phalloidin. A maximum intensity projection along the Z-axis was created for the bottom half of each lumen. The mean pixel intensity of a 625 x 250 μm region of interest centered on the lumen was calculated and subtracted by the mean pixel intensity of a 400 x 100 μm segment of the background of each lumen.

### 2.11 Endothelial permeability assays

After 2 days of culture in the microfluidic devices, 3 μL of either EGM-2 or PAK in EGM-2 was added to the top port of each device. The devices were incubated at 37 °C with 5% CO_2_ for 2 hours. Then, the media in the lumens was replaced with about 4 μL of EGM-2 containing 12.5 μM of 10 kDa fluorescein isothiocyanate (FITC) labeled dextran (FD10S-100MG, Sigma, St. Louis, MO) and imaged immediately. Images were taken in the center Z-plane of each device every 5 seconds for 15 minutes. The videos were transferred to FIJI (ImageJ) for analysis. A vertical line three times the width of the lumen was drawn perpendicular to the lumen and placed at its center. The intensity profile was measured for every pixel on the line at t=0, 5, 10, and 15 minutes. Intensity was normalized to the highest pixel intensity on the t=0 profile for each lumen. Bins of 10 pixels were averaged to smooth the curves.

### 2.12 Neutrophil isolation

All blood samples were obtained according to the institutional review board-approved protocols per the Declaration of Helsinki. Peripheral blood neutrophils were isolated from healthy donors, using a MACSxpress Neutrophil Isolation Kit (130–104-434, Miltenyi Biotec, Bergisch Gladbach, Germany) and a MACSxpress Erythrocyte Depletion Kit (130–098-196, Miltenyi Biotec, Bergisch Gladbach, Germany), according to the manufacturer’s instructions. Informed consent was obtained from donors at the time of the blood draw according to our institutional review board.

### 2.13 Neutrophil extravasation

Isolated neutrophils were resuspended and stained in a 10 nM solution of calcein AM (C3100MP, Thermo Scientific, Waltham, MA) before being resuspended at 7.5 million cells/mL in EGM-2. About 5 μL were loaded into each lumen; then 3 μL of PAK was added to the top port of each device. The devices were imaged immediately following bacterial loading every 10 minutes for 8 hours over a Z-stack of 350 μm with 10 μm steps.

### 2.14 Neutrophil migration

Neutrophil and bacterial loading were carried out as described for extravasation experiments; however, only 20% of the neutrophils were stained to make it easier to distinguish individual cells during data analysis. Images were taken every 30 seconds for 20 minutes at 2-hour intervals over a Z-stack of 80 μm with 10 μm steps.

### 2.15 ROS production

Intracellular ROS production by extravasated neutrophils was measured in the microfluidic devices as reported previously (46). In brief, dihydrorhodamine-123 (DHR-123, D23806, Thermo Fisher Scientific, Waltham, MA) was used to label intracellular ROS. The lumens were preincubated with 10 mM DHR-123 in EGM-2 for 30 minutes at 37 °C. Isolated neutrophils were stained with DiI (V22885, Thermo Fisher Scientific, Waltham, MA) diluted 1:200 in serum-free RPMI (11875119, Thermo Fisher Scientific, Waltham, MA) for 20 minutes at 37 °C. Neutrophils were resuspended at 7.5 million cells/mL in EGM-2 with 10 mM DHR-123. About 5 μL were loaded into each lumen; then 3 μL of PAK was added to the top port of each device. The devices were imaged immediately following bacterial loading every 10 minutes for 8 hours over a Z-stack of 350 μm with 10 μm steps.

### 2.16 Image acquisition

All imaging was performed using a Nikon A1R HD25 Laser Scanning Confocal Microscope built on the Nikon TI2-E Inverted Microscope System, a fully automated stage, and Nikon Elements acquisition software. Stained lumens and confocal reflectance images were captured with a Nikon 20x/0.95 (NA) water immersion objective. Extravasation and ROS experiments were imaged with a Nikon 10x/0.45 (NA) objective. Migration experiments were imaged with a Nikon 20x/0.75 (NA) objective. Extravasation, ROS, and migration experiments were performed at 37 °C and 5% CO_2_ with a cage incubator (H201-T-Unit-BL, Oko Labs, Sewickley, PA).

### 2.17 Image processing and data analysis

Max intensity projections in the Z-axis were created for each extravasation and migration video in Nikon Elements software. Those videos were transferred to FIJI (ImageJ) for data analysis. To eliminate edge effects, neutrophils were analyzed in the center of the device. To analyze extravasated neutrophils, a 690 μm x 345 μm rectangular region of interest defined by the top edge of the lumen in the center of the device was created. The number of neutrophils within this region were counted at each time point. The number of extravasated neutrophils at each time point was normalized to the initial number of neutrophils in the lumen to account for any variability in cell loading. To determine the initial number of neutrophils, a 345 μm x 122.5 μm rectangular region was drawn within the center of the lumen at the first timepoint. The neutrophils within this luminal region were counted.

For migration experiments, extravasated neutrophils that remained in frame through the duration of the 20-minute time lapse and were clearly distinguishable from other cells were tracked using the MtrackJ plugin in FIJI.

### 2.18 MagPix secretion analysis

A multiplexed ELISA was employed to measure secretion levels of inflammatory cytokines in lumen-conditioned media from six different conditions (each collagen concentration with either 3 μL of 5 OD PAK in EGM-2 or EGM-2 alone added to the top port). A MagPix Luminex Xmap system (Thermo Fisher Scientific, Waltham, MA) with a ProcartaPlex Human Inflammation Panel 20Plex (EPX200–12185-901, Invitrogen, Waltham, MA) was used following the manufacturer’s protocol. Lumens were incubated for 8 hours. Media was collected from 6 lumens per replicate, pooled, and centrifuged to remove cellular debris. Data are from 4 replicates. Samples were frozen at -20 °C from the time of collection until the assay was run. Secreted protein levels for each replicate with PAK were normalized from each replicate of the same collagen concentration with EGM-2.

### 2.19 Statistical analysis

For all extravasation, migration, reactive oxygen species, and multiplexed ELISA experiments, data were pooled from three or more independent replicates. Statistical analysis was performed using the estimated marginal means (emmeans) package in R. The statistical analysis accounts for variability within and between replicates and considers independent replicates as individual entities. All collagen concentrations were compared to one another at each time point and analyzed with analysis of variance (ANOVA). For each condition, estimated marginal means and standard errors of the mean (SEM) were calculated and pairwise comparisons were made with Tukey’s adjustment. P-values are labeled as *p<0.05, **p<0.01, ***p<0.001, and ****p<0.0001, unless otherwise noted.

For stained image, rheology, and confocal reflectance microscopy experiments, data were pooled from three or more independent replicates. A one-way ANOVA followed by Tukey’s multiple comparisons test were performed between conditions with an alpha value of 0.05 for the stained image and rheology results. Welch’s ANOVA followed by Games-Howell pairwise comparisons were performed on the confocal reflectance data with an alpha value of 0.05. The mean and SEM were calculated for each condition and P-values are labeled as *p<0.05, **p<0.01, ***p<0.001, and ****p<0.0001, unless otherwise noted.

## 3 Results

### 3.1 Increasing collagen concentration decreases pore size and increases modulus

**Figure 1:**
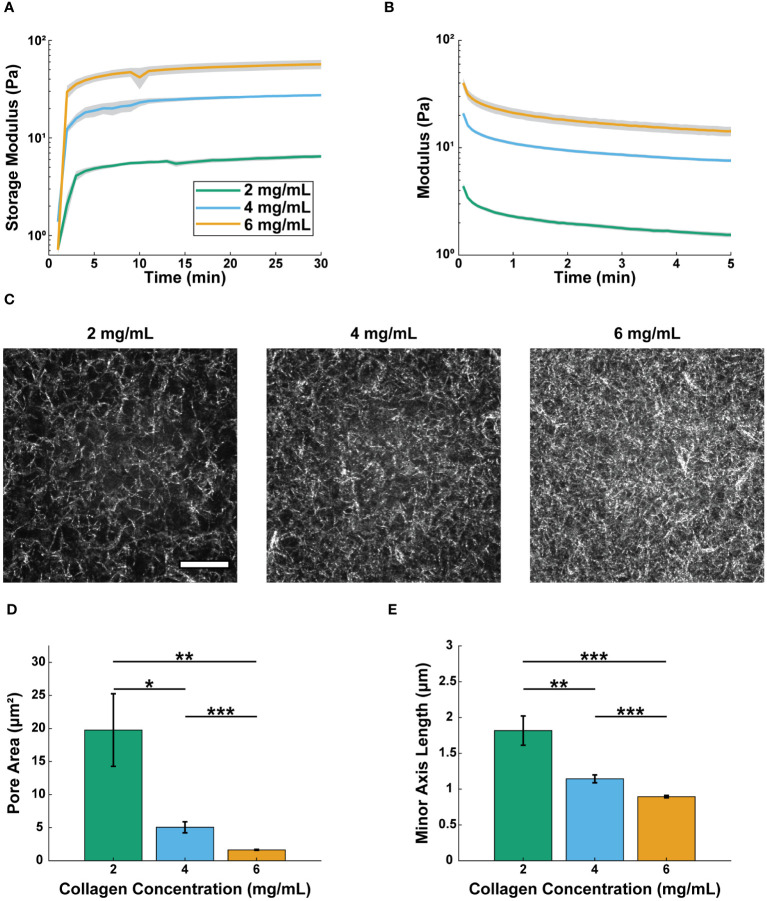
Increasing collagen concentration decreases pore size and increases modulus. Real-time rheological observations for 2, 4, and 6 mg/mL collagen: **(A)** storage moduli (G’), 10 rad/s, 1.0% strain measured at 37 °C and **(B)** stress relaxation 15% strain at 37 °C. For A and B lines show the average modulus (n=3 gels per condition) and the shaded regions show the average modulus ± SEM. One-way ANOVA followed by Tukey’s multiple comparison tests were performed to compare across conditions for the rheological studies. **(C)** Representative confocal reflectance images of 2, 4, and 6 mg/mL collagen (scale bar = 20 μm). Average **(D)** area and **(E)** minor axis length of pores for each concentration (2 mg/mL, n=859 pores; 4 mg/mL, n=2897 pores; 6 mg/mL, n=6586 pores across 4 different gels per condition). For pore size analysis Welch’s ANOVA test was performed followed by Games-Howell pairwise comparisons with an alpha value of 0.05. Error bars indicate mean ± SEM (*p<0.05, **p<0.01, and ***p<0.001).

Neutrophil extravasation and migration are regulated by the structural and mechanical properties of the surrounding ECM (19, 23, 27, 47, 53, 54); therefore, it was important to characterize collagen gels over the range of concentrations used in this study. We created gels with 2, 4, and 6 mg/mL collagen, as this range represents physiologically relevant concentrations throughout various tissues in the body (55). To determine bulk mechanical properties of the collagen gels, we performed oscillatory rheology and found the moduli of the three concentrations were significantly different from each other (**Figure 1A**, **Supplementary Figure S1**). After a 30-minute polymerization period, the Storage Moduli for the 2, 4, and 6 mg/mL collagen gels were 6.4 ± 0.2 Pa, 27.4 ± 0.6 Pa, and 56.7 ± 6.2 Pa, respectively (**Supplementary Tables S1**, **S2**). Following polymerization, stress relaxation tests were conducted (**Figure 1B**). All three collagen gels relaxed to 50% of the original strained modulus 75 seconds after being strained (**Supplementary Tables S1**, **S2**).

We next sought to characterize the microscale organization of the different collagen concentration gels. We used confocal reflectance microscopy to visualize the pore structures of the collagen gels (**Figure 1C**). Pore area and minor axis length (M.A.L.) were determined using Matlab’s erosion function, as has been described previously (52) (**Figures 1D, E**). The pore area of the 2 mg/mL collagen (8.89 ± 2.14 μm^2^) was significantly larger than that of the 4 mg/mL collagen (4.68 ± 0.79 μm^2^), which was significantly larger than the pore area of the 6 mg/mL collagen (0.92 ± 0.05 μm^2^). The same trends were seen for pore M.A.L. (**Supplementary Table S2).** Together, these data show that increasing collagen concentration increases the modulus of the gels, while also decreasing pore size. However, changing the collagen concentration over the selected range did not affect the viscoelastic nature of the gels.

### 3.2 Collagen concentration does not alter vessel formation or barrier function

**Figure 2:**
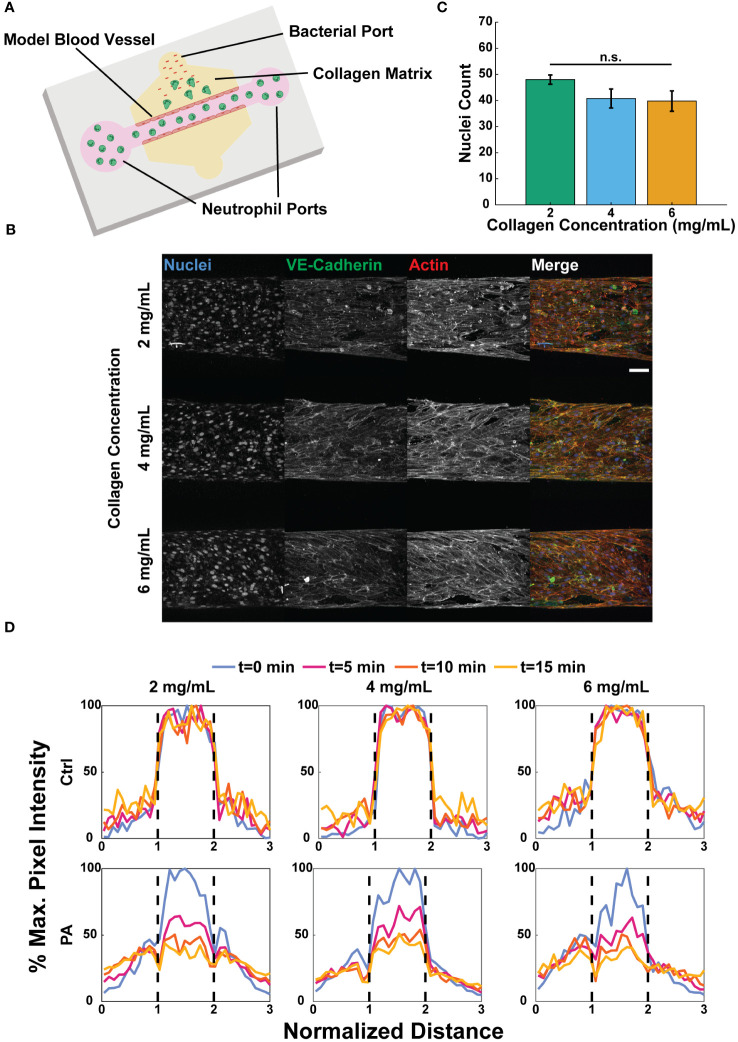
Collagen concentration does not alter vessel formation or barrier function. **(A)** Schematic of the microfluidic system used to model tissue infection near a blood vessel with varied collagen concentrations. **(B)** Representative maximum intensity projections of confocal images of HUVECs seeded in microfluidic devices with 2, 4, and 6 mg/mL collagen and stained with Hoechst (nuclei, blue), anti-VE-cadherin (tight junctions, green), and phalloidin (actin, red) (scale bar = 100 μm). **(C)** Number of nuclei counted in a fixed region of interest about the bottom of each lumen (n=4 per collagen concentration). The three collagen concentration conditions were compared to each other via one-way ANOVA followed by Tukey’s multiple comparisons test were performed between conditions with an alpha value of 0.05. Error bars indicate mean ± SEM. **(D)** Representative graphs of FITC-dextran diffusion at 0, 5, 10, and 15 minutes. EGM-2 (ctrl) or *P. aeruginosa* in EGM-2 (PA) was added to the top bacterial port 2 hours prior to adding FITC-dextran to the lumens. Pixel intensity normalized against the maximum pixel intensity at time t=0 min. Dashed lines indicate the edges of each lumen.

We previously developed a microphysiological system with relevant components of a tissue microenvironment, including a model blood vessel surrounded by extracellular matrix (ECM) proteins to quantify the human neutrophil response to infection (45). Here, we used that model to investigate the effect of collagen concentration on the neutrophil response. To simulate an infection, neutrophils were loaded into the lumen of the vessel, and bacteria were added to the surrounding matrix via a port at the top of the device (**Figure 2A**). For this study, we used type I collagen gels as our model ECM, as this is the most abundant protein in tissue matrices (17).

The barrier function of endothelial cells, which has been linked to neutrophil activation, has been shown to increase with increasing stiffness of the underlying substrate on which they are seeded (40, 56). Therefore, we sought to determine if collagen concentration affected the formation or barrier function of endothelial lumens. To determine if collagen concentration affects cell density in the lumens, confluent monolayers were formed with human umbilical vein endothelial cells (HUVECs) in each collagen concentration, and endothelial nuclei (Hoechst), actin (phalloidin), and tight junctions (anti-VE-cadherin) were stained (**Figure 2B**). Counting the number of nuclei in a fixed region of interest revealed no significant differences in the number of cells per area across the three conditions (**Figure 2C**, **Supplementary Table S2**). Collectively, these results suggest that there was no significant difference in endothelial cell loading or morphology in the different collagen concentration substrates.

During homeostasis endothelial cells form tight junctions to prevent vascular leakage; however, upon infection the cells permit the passage of inflammatory markers between the ECM and the lumen to aid in neutrophil activation and extravasation (32). Therefore, we tested the barrier function of the lumens in the three collagen concentrations using a FITC-dextran to visualize vascular permeability. *P. aeruginosa* was added to the top ports of devices to simulate an infection. FITC-dextran in medium was added to the lumens 2 hours after adding either medium alone or *P. aeruginosa* in medium to the top bacterial ports of each device, and diffusion into the surrounding ECM was visualized for 15 minutes. Representative graphs show the percentage of the maximum fluorescence intensity, relative to t=0, at t=0, 5, 10, and 15 minutes across a region three lumen diameters in length and centered around each lumen (**Figure 2D**, **Supplementary Figure S2A, B**). In each collagen concentration, the endothelial cells retained the FITC in the absence of *P. aeruginosa*, indicating significant barrier function. However, in the presence of *P. aeruginosa* the endothelial lumens became leaky, permitting dextran to diffuse into the ECM.

Upon inflammatory challenge luminal endothelial cells upregulate the expression of surface proteins, such as ICAM-1, to facilitate neutrophil extravasation (32, 33, 57). To determine if endothelial cells produce ICAM-1 in our system, we added *P. aeruginosa* to the top bacterial port of lumen devices and allowed them to incubate for 2 hours before fixing and staining the endothelia for nuclei (Hoechst), actin (phalloidin), and ICAM-1 (anti-ICAM-1) (**Supplementary Figure S2C**). No significant differences in ICAM-1 were observed by endothelial lumens in each of the 2, 4, and 6 mg/mL collagen conditions (**Supplementary Figure S2D**, **Supplementary Table S2**).

### 3.3 Neutrophils exhibit enhanced extravasation into medium-density collagen

**Figure 3:**
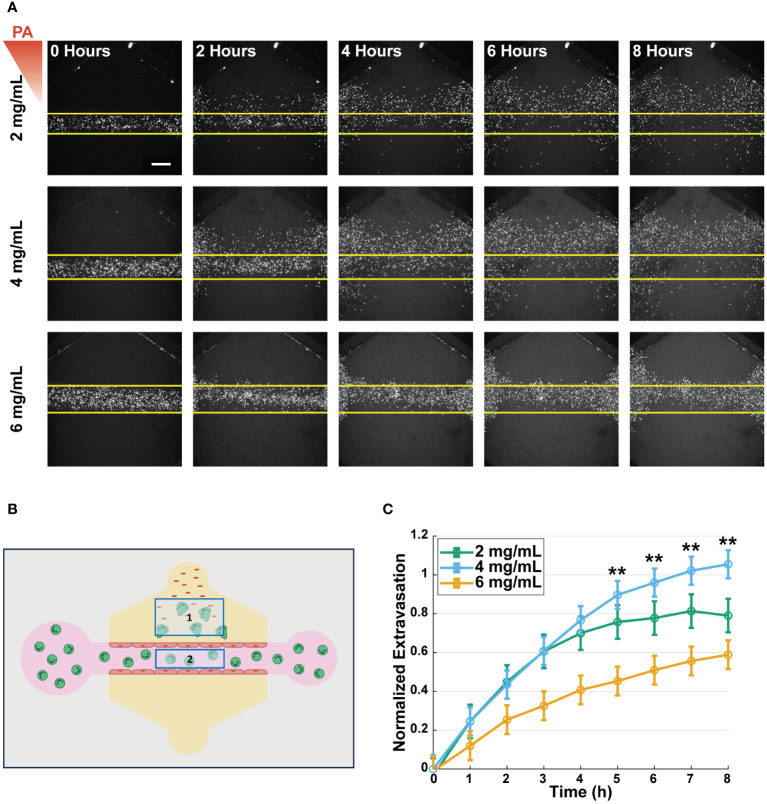
Neutrophils exhibit enhanced extravasation in medium-density collagen. **(A)** Representative images of extravasated neutrophils in 2, 4, and 6 mg/mL collagen at 0, 2, 4, 6, and 8 hours after stimulation with *P. aeruginosa* (scale bar = 250 μm). Yellow lines indicate the edge of the lumen. The red triangle shows the initial bacterial gradient. **(B)** Schematic of the analysis scheme used to determine normalized extravasation. Neutrophils counted in Box 1 at all timepoints are divided by number of neutrophils counted in Box 2 at t=0 to normalize against the initial number of cells loaded into each lumen. **(C)** Normalized number of extravasated neutrophils in microfluidic devices with 2, 4, and 6 mg/mL collagen. Data quantified from 14 devices (2 mg/mL), 20 devices (4 mg/mL), and 19 devices (6 mg/mL) across 5 independent experiments and 5 neutrophil donors. All collagen concentrations were compared to each other at each time point. For each condition, estimated marginal means and SEM were calculated and pairwise comparisons were performed with Tukey’s adjustment. Error bars indicate estimated marginal means ± SEM. Asterisks indicate significance between the 4 and 6 mg/mL condition at the same timepoint where **p<0.01.

After characterizing the collagen networks and confirming no changes in lumen structure or barrier function in the various collagen concentrations, we sought to study the effect of collagen concentration on neutrophil extravasation, as this is the first step in the neutrophil response to infection. To quantify extravasation, primary human neutrophils were isolated from whole blood samples, stained with a fluorescent marker (calcein AM), and loaded into the lumens. Extravasation was then induced by loading *P. aeruginosa* into the top bacterial port of each device. Confocal microscopy was used to image neutrophil extravasation for 8 hours (**Figure 3A**). Extravasated neutrophils were quantified as the number of extravasated neutrophils in a region of interest (ROI) outside of the lumen divided by the number of neutrophils in a second ROI inside the lumen at t=0 to normalize for any discrepancies in neutrophil loading (**Figure 3B**). Significantly more neutrophils extravasated into the 4 mg/mL collagen than in the 6 mg/mL collagen 5–8 hours after *P. aeruginosa* stimulation. Neutrophil extravasation in 2 mg/mL collagen was greater than in 6 mg/mL collagen but less than in 4 mg/mL collagen; however, the differences did not reach statistical significance (**Figure 3C**). This extravasation is dependent on the presence of an inflammatory stimulus as no extravasation occurs in 2, 4, or 6 mg/mL collagen in response to EGM-2 alone (**Supplementary Figure S3**).

Given the mechanosensitive nature of the endothelial cells and their role in the activation of neutrophils, we wanted to determine if the differences in neutrophil extravasation were due to the collagen concentration affecting the neutrophils directly or indirectly through interactions with the endothelial cells. Therefore, we repeated the extravasation experiments in the absence of an endothelium (**Supplementary Figure S4**). We found no significant differences in the number of extravasated neutrophils across the 2, 4, and 6 mg/mL collagen conditions. Together these data show that there is greater neutrophil extravasation into the 4 mg/mL collagen compared to the 6 mg/mL collagen and imply that this difference is due to an endothelial cell-dependent mechanism.

### 3.4 Increasing collagen concentration decreased neutrophil migration speed and distance

**Figure 4:**
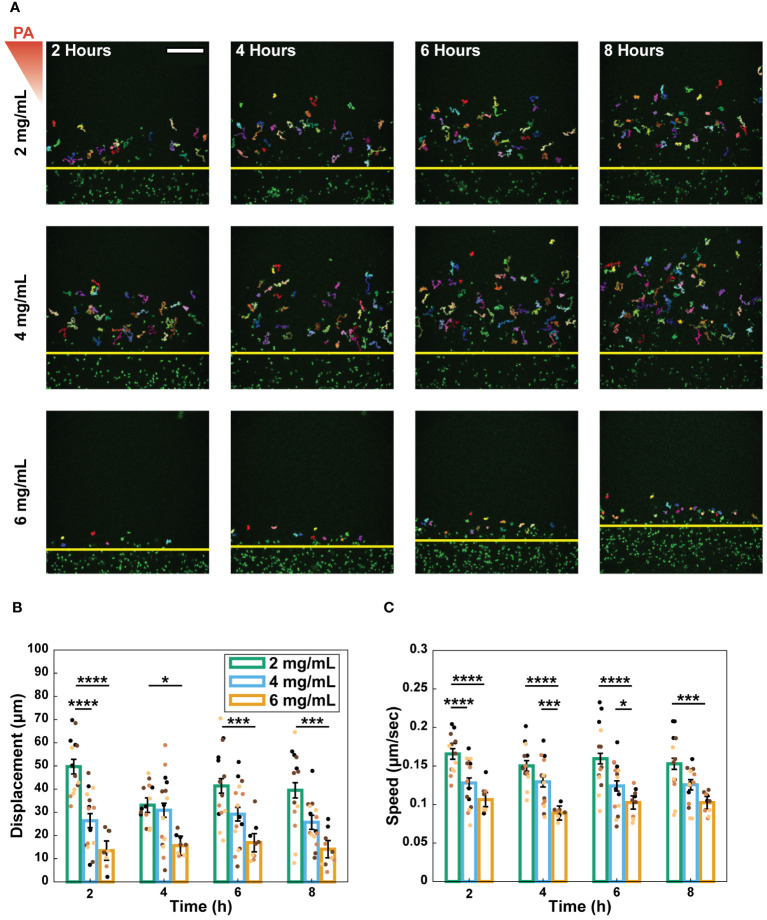
Increasing collagen concentration decreased neutrophil migration speed and distance. **(A)** Representative images and tracks of migrating neutrophils in 2, 4, and 6 mg/mL collagen at 2, 4, 6, and 8 hours after stimulation with *P. aeruginosa* (scale bar = 250 μm). Each individual track is shown in different colors. Yellow lines indicate the edge of the lumen. The red triangle shows the initial bacterial gradient. Migration properties: **(B)** displacement and **(C)** speed of neutrophils quantified over 20-minute increments at 2, 4, 6, and 8 hours after stimulation with *P. aeruginosa*. Extravasated neutrophils, from 13 devices (2 mg/mL), 15 devices (4 mg/mL), and 10 devices (6 mg/mL) across 4 independent experiments and 4 neutrophil donors, were tracked using MTrackJ in Fiji. Dots indicate the average migration property for all neutrophils in each device and the dot colors indicate different independent experiments. All collagen concentrations were compared to each other at each time point. For each condition, estimated marginal means and SEM were calculated and pairwise comparisons were performed with Tukey’s adjustment. Error bars indicate the estimated marginal means ± SEM. Asterisks indicate significance between conditions at a given timepoint (*p<0.05, ***p<0.001, and ****p<0.0001).

Following extravasation, neutrophils must navigate through the ECM to respond to infection. To determine the effect of collagen concentration on this next step of the neutrophil response, we quantified the migration properties of extravasated neutrophils in the different collagen matrices. Extravasated neutrophils were imaged every 30 seconds for 20-minutes 2, 4, 6, and 8 hours after *P. aeruginosa* stimulation (**Figure 4A**). We calculated the net displacement as the distance between the starting and ending positions for each 20-minute interval (**Figure 4B**). Migration path length was measured for each neutrophil and was used with displacement to determine the straightness of migration: the path length of a neutrophil divided by its net displacement (**Supplementary Figure S5**). Lastly, speed was computed as the total path length travelled by a neutrophil divided by the time elapsed during the track (**Figure 4C**).

Generally, migration speed, displacement, and path length significantly decreased with increasing collagen concentration (**Figures 4B, C**, **Supplementary Figure S5B**). This trend was observed for straightness at the 2-hour time point; however, it did not persist at later timepoints (**Supplementary Figure S5A**).

To determine if these differences were due to direct neutrophil-collagen interactions or indirect interactions involving the endothelium, migration experiments were repeated in the absence of an endothelium (**Supplementary Figure S6**). Neutrophil migration was not reported beyond the 2-hour timepoint for conditions without an endothelium as most neutrophils do not survive past 2 hours without the pro-survival cytokine granulocyte macrophage colony stimulating factor (GM-CSF) (45). Without an endothelium, neutrophils in the 4 mg/mL collagen migrated faster and farther than neutrophils in the 2 mg/mL collagen (**Supplementary Figures S6B, D**). However, straightness was not significantly different (**Supplementary Figure S6E**). Together these results suggest that the observed differential migration is, at least in part, regulated by an endothelial cell-dependent mechanism.

### 3.5 Infection and collagen concentration affect endothelial secretion of inflammatory markers

**Figure 5:**
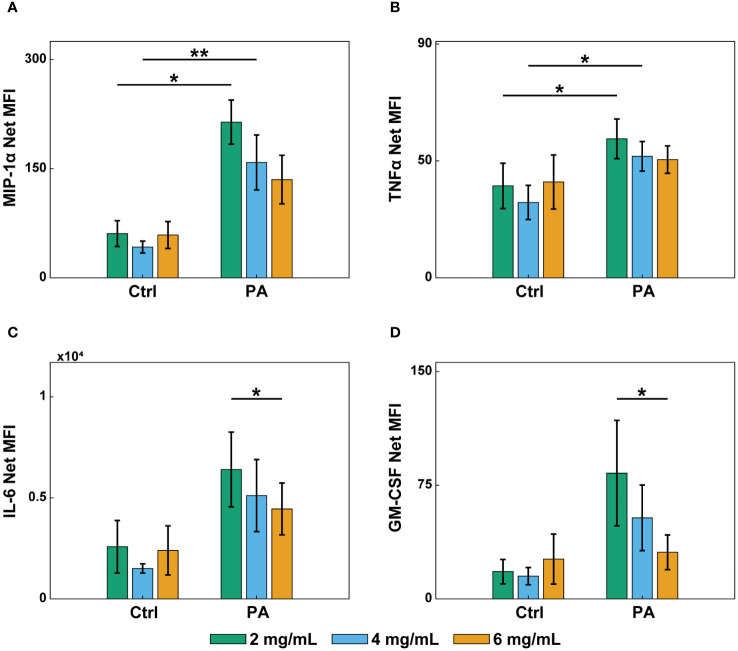
Infection and collagen concentration affect endothelial secretion of inflammatory markers. Multiplexed ELISA of conditioned media from lumens in 2, 4, and 6 mg/mL collagen devices treated with EGM-2 (Ctrl) or *P. aeruginosa* in EGM-2 (PA) for 8 hours. Conditioned media was collected from 6 devices per condition and pooled in 4 independent experiments. Samples were analyzed with a Luminex Magpix device and a ProcartaPlex Human inflammation Panel, 20Plex. Graphs show net mean fluorescence intensity (MFI) of soluble inflammatory markers: **(A)** MIP-1α, **(B)** TNFα, **(C)** IL-6, and **(D)** GM-CSF. All collagen concentrations with and without *P. aeruginosa* were compared to each other for each inflammatory marker. For each condition, estimated marginal means and SEM were calculated and pairwise comparisons were performed with Tukey’s adjustment. Error bars indicate estimated marginal means ± SEM. Asterisks indicate significance between conditions for a give cytokine (*p<0.05 and **p<0.01).

During an infection, endothelial cells secrete inflammatory markers that recruit and activate neutrophils circulating in the blood (58). To determine if the differential neutrophil extravasation and migration were due to changes in endothelial secretion profiles of inflammatory markers, a multiplexed ELISA was performed. Endothelial lumens were formed in 2, 4, and 6 mg/mL collagen. Devices were treated with media or *P. aeruginosa* and incubated for 8 hours. Conditioned media was then collected from inside the lumens, and a ProcartaPlex Human Inflammation Panel was used to determine the net mean fluorescence intensity (MFI) of 20 select pro- and anti-inflammatory signaling molecules (**Figure 5**, **Supplementary Figure S7**). Secretion of key inflammatory markers (MIP-1α, TNFα) increased in the 2 and 4 mg/mL conditions when stimulated with *P. aeruginosa* compared to controls (**Figures 5A, B**). Furthermore, IL-6 and GM-CSF, which have previously been reported to increase neutrophil extravasation and lifetime, respectively (45), were increased in the 2 mg/mL compared to the 6 mg/mL collagen in the presence of *P. aeruginosa* (**Figures 5C, D**). These results suggest that altered endothelial secretion of inflammatory markers, such as IL-6 and GM-CSF may contribute to the observed differences in neutrophil migration. However, none of the secretion profiles match the trends seen in extravasation, suggesting that the differences in extravasation are not due to secreted factors by the endothelium (**Figures 3**, **5**, **Supplementary Figure S7**).

### 3.6 Percentage of extravasated neutrophils producing ROS is unaffected by collagen concentration

**Figure 6:**
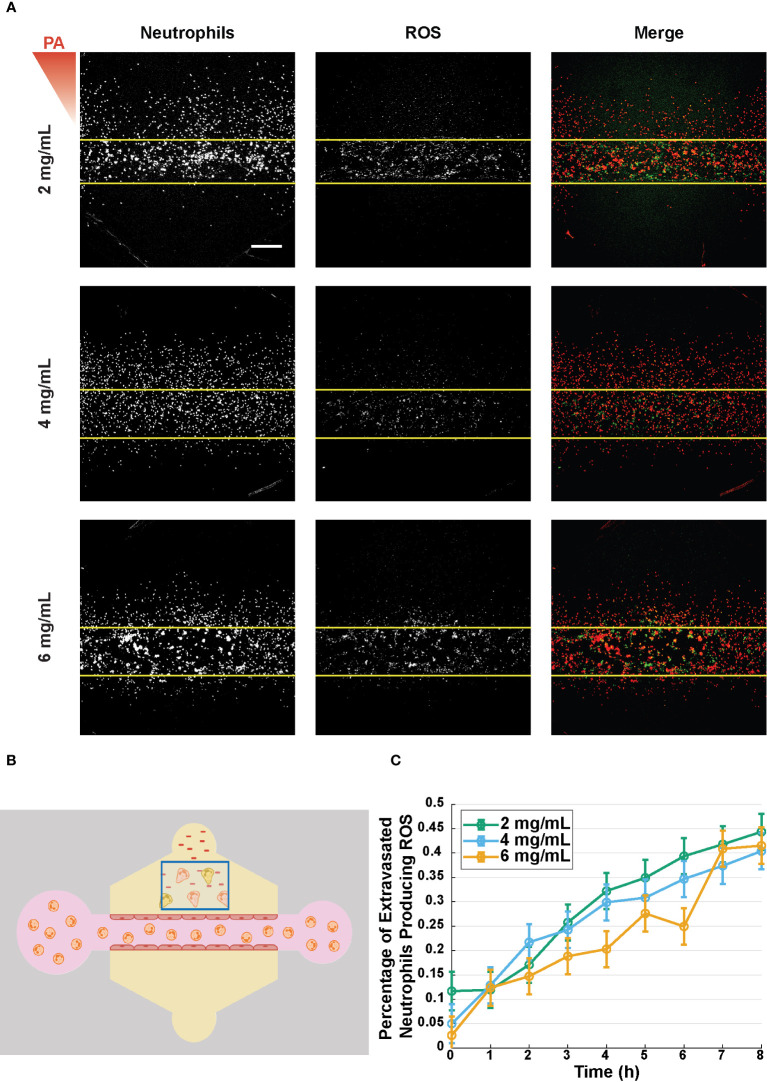
Percentage of extravasated neutrophils producing ROS is unaffected by collagen concentration. **(A)** Representative reactive oxygen species (ROS) production in microfluidic devices with 2, 4, and 6 mg/mL collagen 8 hours after stimulation with *P. aeruginosa*. From left to right the images show: neutrophils stained with DiI (red), dihydrorhodamine-123 (DHR-123, green), and a merged image showing both the neutrophils and rhodamine (scale bar = 250 μm). The red triangle shows the initial bacterial gradient. **(B)** Schematic for quantifying the percent of extravasated neutrophils producing ROS. Extravasated neutrophils in the indicated box that produce ROS with overlapping red and green signals are divided by the total number of neutrophils in the box. **(C)** Percentage of extravasated, ROS producing neutrophils in microfluidic devices with 2, 4, and 6 mg/mL collagen. Data quantified from 16 devices per condition across 4 independent experiments and 4 neutrophil donors. All collagen concentrations were compared to each other at each time point. For each condition, estimated marginal means and SEM were calculated and pairwise comparisons were performed with Tukey’s adjustment. Error bars indicate estimated marginal means ± SEM.

Once neutrophils reach the site of infection, they employ a variety of antimicrobial functions to eliminate pathogens, and one of the primary functions is the production of reactive oxygen species (ROS). It has been shown previously that neutrophil binding to collagen, via β1 integrins on the neutrophil surface, facilitates ROS production (29–31). Thus, to determine if ROS production was altered by the surrounding collagen concentration, a ROS indicator (DHR-123) was used to measure the percentage of ROS-producing neutrophils following extravasation (**Figure 6A**). The percentage of ROS-producing neutrophils was calculated by dividing the number of rhodamine positive neutrophils in a ROI outside the lumen by the total number of neutrophils in that ROI (**Figure 6B**). No significant differences in percentage of ROS-producing neutrophils were seen across the three collagen concentrations over the 8-hour observation period (**Figure 6C**).

## 4 Discussion

In this study, we used a physiologically relevant microfluidic device to investigate the effects of collagen concentration on neutrophil extravasation, migration, and ROS production. Our data showed greater neutrophil extravasation into 4 mg/mL compared to 6 mg/mL collagen in response to *P. aeruginosa* in the presence of an endothelium. However, in the absence of endothelium, no differences in the number of extravasated neutrophils were observed across collagen concentrations. Further, we found that increasing collagen concentration, which decreased pore size and increased modulus, decreased neutrophil migration speed and distance. In the absence of an endothelium, neutrophils in 4 mg/mL collagen migrated faster and farther than those in 2 mg/mL collagen. Lastly, no differences in the percentage of neutrophils producing ROS were observed in the extravasated neutrophils in various collagen concentrations. Collectively, these results show that collagen concentration affects neutrophil extravasation and migration in an endothelial cell-dependent manner, potentially due to differential activation of the endothelium. It is possible that the structural differences in collagen matrices contribute to changes in endothelial activation, neutrophil extravasation, and neutrophil migration.

Previous studies have shown that changes to the underlying substrate influence endothelial phenotype and function. Lugo-Cintron, et al. recently found that high collagen concentration can alter vessel behavior, as measured by the number of lymphatic endothelial nuclei per area in a similar microfluidic system (48). Changes to the number of cells per area in the lumen would affect the number of available sites for neutrophil extravasation and could alter the total protein expression of inflammatory markers. Our results showed no change in the number of nuclei per area across the collagen concentrations after a 2-day incubation. This may be due to our shorter culture time in the devices before starting our extravasation experiments or difference in endothelial cell types used in the two studies. Additionally, Huynh, et al. demonstrated that endothelial cells exhibit increased permeability when seeded on high stiffness matrices compared to softer matrices (40). Furthermore, they showed that the increased permeability allowed for enhanced leukocyte extravasation. Thus, we tested the permeability of lumens in each of the collagen conditions and found no notable differences. Although the storage moduli for each concentration was significantly different than the others, the gels in our study were orders of magnitude softer than those used by Hyunh, et al. Furthermore, their transmigration experiments were conducted with endothelial cells seeded on polyacrylamide gels, as opposed to the collagen matrices used in our study. These differences in their system compared to ours may explain the discrepancy in the results.

It is unlikely that the increased extravasation into 4 mg/mL collagen compared to 6 mg/mL collagen was solely due to the neutrophil interactions with the collagen, given the lack of differences in extravasation without an endothelium. Endothelial cell stiffness is regulated by the stiffness of the underlying matrix and increases in stiffness lead to enhanced clustering of adhesion molecules that bind neutrophils, like ICAM-1 (38). Therefore, it would be expected that increasing collagen concentration would increase neutrophil extravasation. Interestingly, that was not observed in this study. However, Wang, et al. demonstrated in mice that neutrophils find pockets of low protein expression in the basement membranes on the tissue side of the endothelium as ideal spots for extravasation (59). These competing factors indicate there may be an optimal collagen concentration for neutrophil extravasation. If the matrix is too soft then there is less clustering and activation of adhesion molecules on the endothelium, but if the matrix is too dense then there are limited spaces of low protein expression where the neutrophils can push into the network. Thus, it may be the case that the matrix underlying the endothelium in the 6 mg/mL collagen is too dense to permit neutrophils to extravasate, while the 4 mg/mL provides suitable pockets for extravasation while maintaining sufficient stiffness, even though it has a lower modulus than the 6 mg/mL collagen.

Following extravasation out of the blood vessel, neutrophils must migrate through the ECM to reach sites of inflammation. We found that, following extravasation through an endothelium, increasing collagen concentration decreased neutrophil migration speed and distance. While structural proteins such as type I collagen provide scaffolding to facilitate mesenchymal cell migration, neutrophils can employ amoeboid migration in 3-dimensional environments such as the ECM and navigate the ECM in an integrin-independent manner (60–63). In this mode of migration, neutrophils are primarily limited by the pore structure of their environment. The lobular design of neutrophil nuclei allows them to migrate through small pores; however, when the pores of the ECM approach the size of these nuclear lobes, neutrophil migration slows considerably. It was previously shown that neutrophil migration arrests when the cross-sectional pore area approaches 2 μm^2^ (27). We hypothesize that the differences in neutrophil migration we observed were significantly influenced by the pore sizes of the various collagen concentration matrices, especially in the case of the 6 mg/mL collagen, as the average pore sizes were less than 2 μm^2^. However, future studies isolating the gel porosity should be conducted to determine if this is indeed the cause of differences in migration. In the absence of an endothelium, neutrophils migrated faster in the 4 mg/mL collagen than in the 2 mg/mL collagen, suggesting that the neutrophil-endothelial cell interaction might influence the subsequent neutrophil migration in gels with sufficiently large pores.

Jeon et al. previously demonstrated that substrate stiffness can alter endothelial secretion levels of various cytokines, including MCP-1, IL-4, and IL-3 (41). Therefore, we investigated how collagen concentration altered endothelial secretion profiles of common inflammatory markers. We found that the presence of *P. aeruginosa* generally increased the level of pro-inflammatory markers, as expected, and collagen concentration affected the secretion of IL-6 and GM-CSF, with more secretion seen at lower collagen concentrations. This was interesting, as we have previously shown that IL-6 and GM-CSF mediate neutrophil extravasation and lifetime, respectively (45). Therefore, it is possible that the increased levels of these two cytokines in the 2 mg/mL collagen condition compared to the 6 mg/mL collagen condition may contribute to the differences in neutrophil migration. Future studies are needed to determine the effects of IL-6 and GM-CSF on neutrophil migration in 3D collagen matrices.

Previous studies reported that neutrophil binding to type 1 collagen via β1 integrin increased ROS production of neutrophils adhered to 96-well plates (29). However, over the course of the first 8 hours after stimulation with *P. aeruginosa* we did not observe significant differences in the percentage of ROS-producing neutrophils. It is possible that ROS production in 3D matrices, like migration, is carried out primarily through integrin-independent mechanisms.

Collectively, our results show that collagen concentration, over the range tested (2–6 mg/mL), regulates neutrophil extravasation and migration in response to an infection, likely through direct and indirect mechanisms. These findings bring about interesting mechanistic questions. Specifically, we found differential neutrophils extravasation across the collagen concentrations in the presence of an endothelium. Yet there were no significant differences in the level of secreted inflammatory markers that matched this observation. It is possible that there is a time-dependent aspect of the inflammatory marker secretion; however, these differences would not be captured by our data as the multiplexed ELISA only provides protein levels at a single timepoint. Additionally, the ELISA does not capture expression levels of endothelial surface proteins that play pivotal roles in neutrophil activation, such as selectins and adhesion molecules. Furthermore, it is known that endothelial cells produce laminin-rich basement membranes (64). However, the extent of this matrix deposition in the different collagen concentration devices and its effect on the local modulus and pore structure near the endothelium is unknown. Future studies should investigate the potential roles of endothelial surface protein expression and basement membrane deposition on the neutrophil response. Lastly, type I collagen gels are often used to model the ECM, as it is the most abundant protein in the network; however, there are many other proteins present in the ECM that interact with neutrophils, such as fibronectin, fibrinogen, laminin, and other collagens (17). Future studies should seek to examine the effects of other matrix proteins on the neutrophil infectious response.

It is increasingly appreciated that neutrophil dysfunction contributes to the pathogenesis of diverse diseases and conditions. As such, there is growing interest in targeting neutrophils for therapeutic treatments of those diseases. To effectively target neutrophils, it is imperative to understand the factors that regulate their response to inflammation and infection. In this study we showed that collagen concentration plays a regulatory role in the neutrophil response to infection. Further, we demonstrated the value of using microphysiological systems to investigate regulatory mechanisms of neutrophil biology that are inaccessible to traditional *in vitro* and *in vivo* models. Continuing use and improvement of such systems will allow us to deepen our understanding of the fundamental biology of neutrophils, thereby facilitating more efficient development of therapeutics.

## Data availability statement

The raw data supporting the conclusions of this article will be made available by the authors, without undue reservation.

## Ethics statement

The studies involving humans were approved by University of Colorado Institutional Review Board. The studies were conducted in accordance with the local legislation and institutional requirements. The participants provided their written informed consent to participate in this study.

## Author contributions

CC: Conceptualization, Data curation, Formal Analysis, Funding acquisition, Investigation, Methodology, Project administration, Resources, Supervision, Validation, Visualization, Writing – original draft, Writing – review & editing. TP: Data curation, Investigation, Writing – review & editing. MP: Data curation, Investigation, Writing – review & editing. NW: Data curation, Investigation, Writing – review & editing. LH: Conceptualization, Funding acquisition, Methodology, Project administration, Resources, Supervision, Validation, Writing – original draft, Writing – review & editing.
