# Supplementary material for: Collagen concentration regulates neutrophil extravasation and migration in response to infection in an endothelium dependent manner
Source: Front Immunol. 2024 Jul 3;15:1405364. doi: 10.3389/fimmu.2024.1405364 (PMC11251947; doi:10.3389/fimmu.2024.1405364)
Supplement: Supplementary file 1 [file DataSheet_1.pdf]

Supplementary Material

1 Supplementary Figures and Tables

Supplemental Table 1: Rheological characterization of collagen gels. Storage modulus and time to 50% stress relaxation in 2, 4, and 6 mg/mL collagen gels. Values represent the mean ± SEM (*p<0.05 for 2 vs 4 mg/mL, †p<0.001 for 2 vs 6 mg/mL, and #p<0.01 for 4 vs 6 mg/mL).

**Collagen Concentration**	**Storage Modulus (Pa)**	**Time to 50% Relaxation (sec)**
2 mg/mL	$6.4 \pm 0.2^{*\dagger}$	74.9 ± 11.0
4 mg/mL	$27.4 \pm 0.6^{*\#}$	76.0 ± 1.1
6 mg/mL	$56.7 \pm 6.2^{\dagger\#}$	74.6 ± 0.9

Supplemental Table 2: P-values for various comparisons between 2, 4, and 6 mg/mL collagen. P-values for comparisons between 2, 4, and 6 mg/mL collagen conditions for rheology, confocal reflectance, and stained image experiments.

Comparisons	Storage Modulus	Time to 50% Relaxation	Pore Area	Pore Minor Axis Length	Nuclei Count	ICAM-1 Expression
2 vs 4 mg/mL	P=0.0143	P=0.3581	P=0.0222	P=0.0043	P= 0.3029	P=0.93767
2 vs 6 mg/mL	P=0.0001	P=0.4434	P=0.0029	P=0.0002	P= 0.2248	P=0.91051
4 vs 6 mg/mL	P=0.0028	P=0.9801	P= 0.0003	P=0.0002	P= 0.9743	P=0.73957

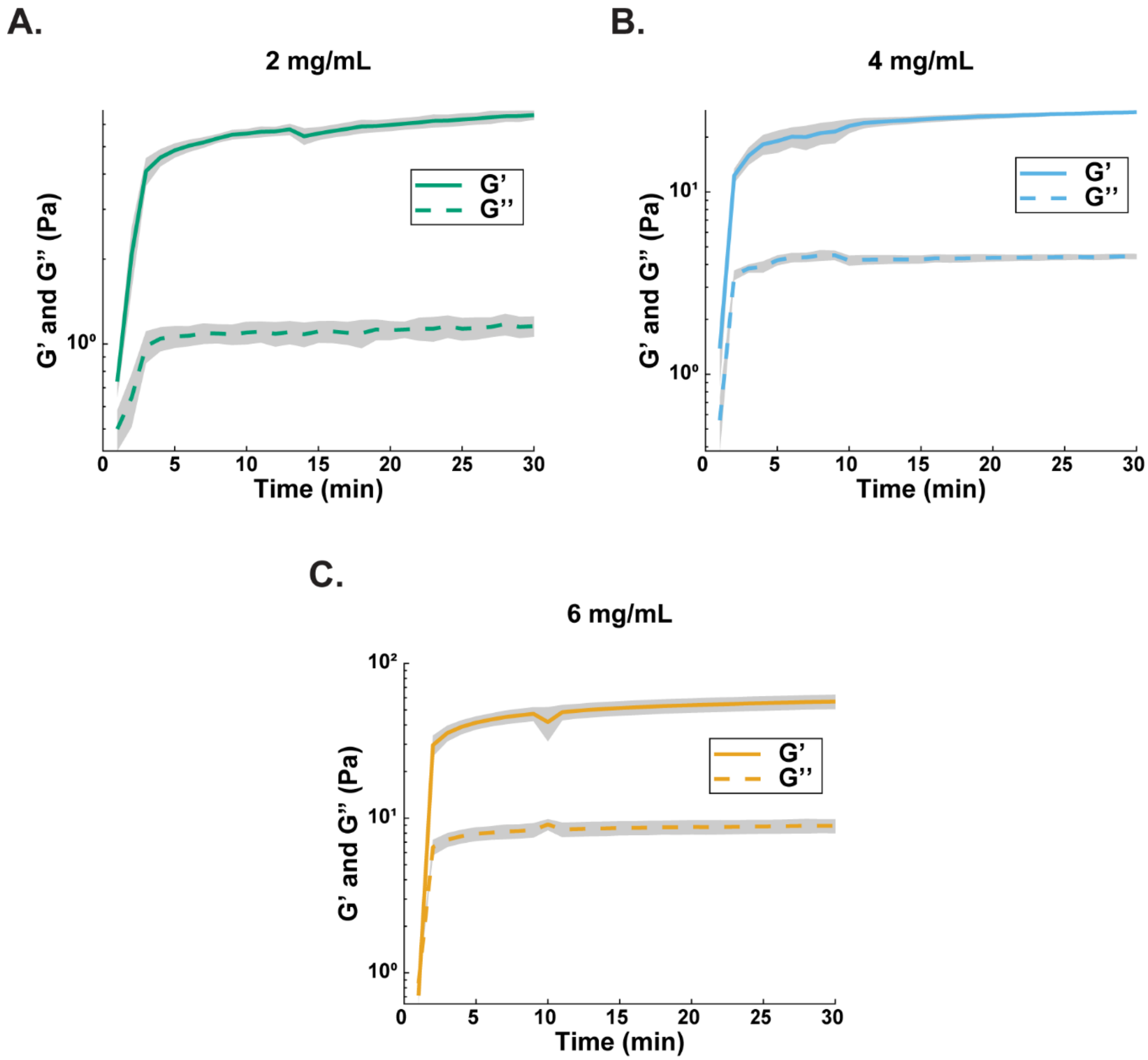

Supplementary Figure 1: Storage and loss moduli graphs for 2, 4, and 6 mg/mL collagen gels. Real-time rheological observations of storage (G'') and loss (G') moduli (10 rad/s, 1.0%) for **(A)** 2 mg/mL, **(B)** 4 mg/mL, and **(C)** 6 mg/mL collagen measured for 30 minutes at 37 ºC. Lines show the average modulus (n=3 gels per condition) and the shaded regions show the average modulus ± SEM.

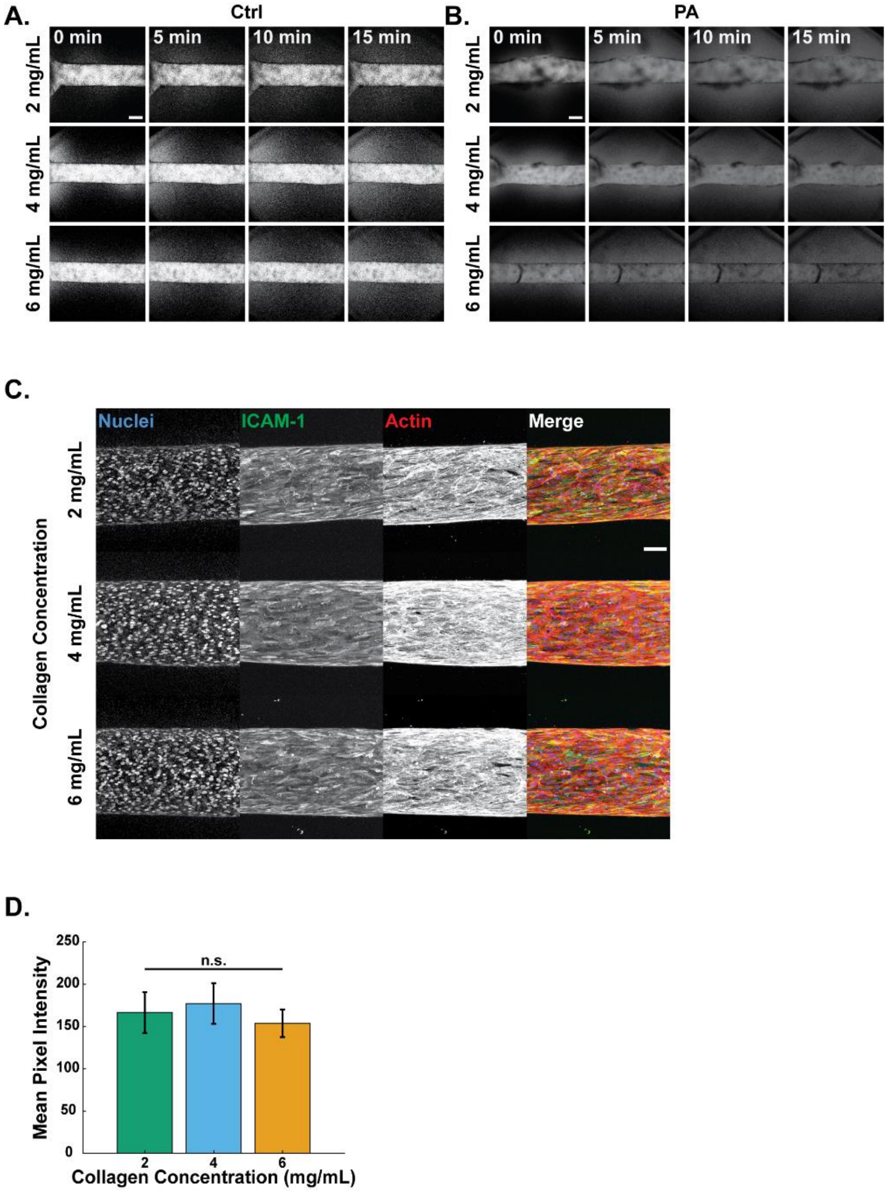

Supplementary Figure 2: Permeability images and ICAM-1 stains for 2, 4, and 6 mg/mL collagen lumens. Representative images of FITC-dextran diffusion at 0, 5, 10, and 15 minutes. **(A)** EGM-2 (Ctrl) or **(B)** *P. aeruginosa* in EGM-2 (PA) was added to the top bacterial port 2 hours prior to adding FITC-dextran to the lumens (scale bar = 250 μm). **(C)** Representative maximum intensity projections of confocal images of HUVECs seeded in microfluidic devices with 2, 4, and 6 mg/mL collagen and stained with Hoechst (nuclei, blue), anti-ICAM-1 (ICAM-1, green), and phalloidin (actin, red) (scale bar = 100 μm). **(D)** Mean fluorescence intensity of ICAM-1 in a fixed region of interest about the bottom of each lumen (n=3 per collagen concentration). The three collagen concentration conditions were compared to each other via one-way ANOVA followed by Tukey's multiple comparisons test were performed between conditions with an alpha value of 0.05. Error bars indicate mean ± SEM.

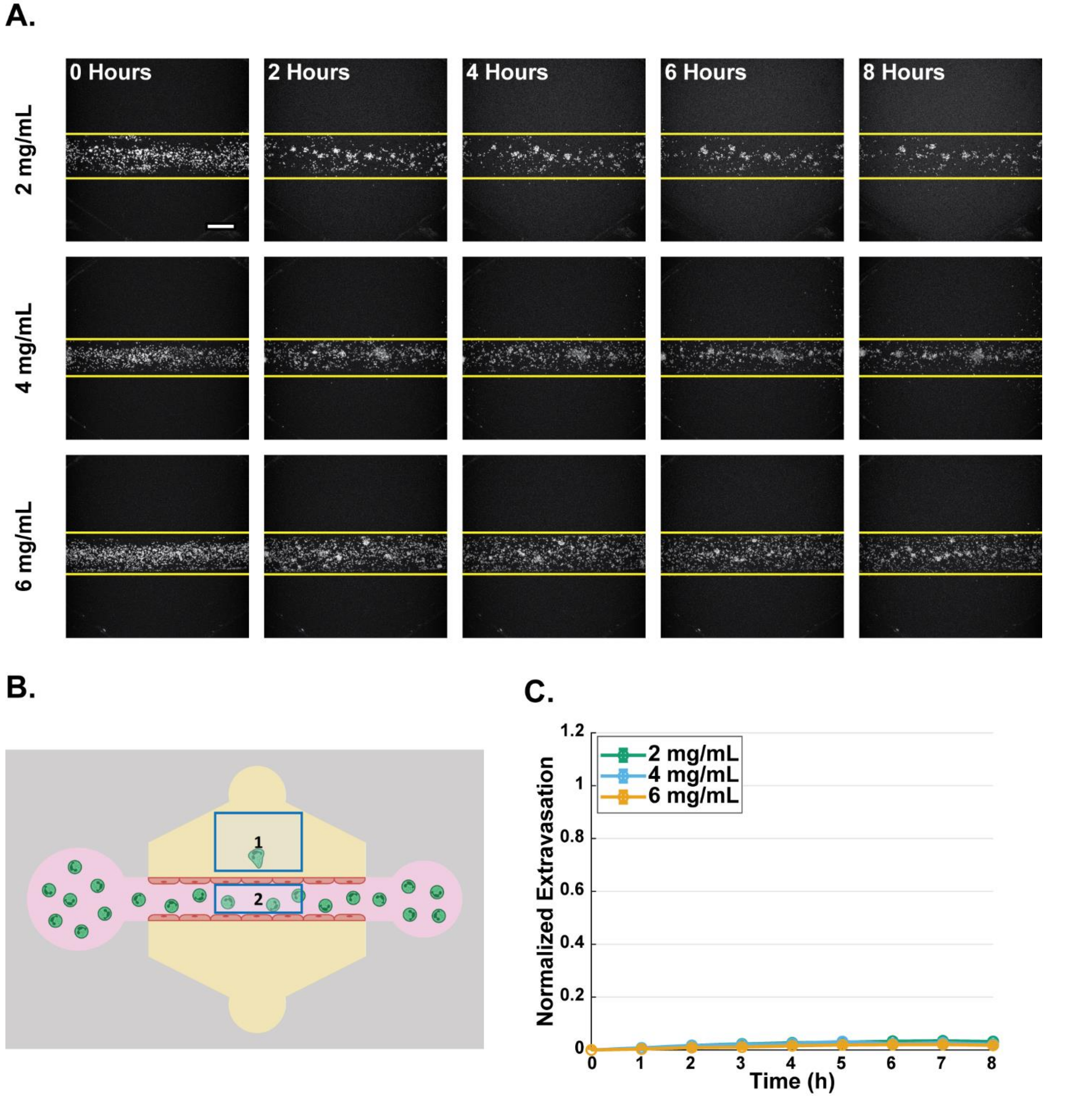

Supplementary Figure 3: Neutrophils do not extravasate in the absence of *P. aeruginosa*. (A) Representative images of extravasated neutrophils in 2, 4, and 6 mg/mL collagen at 0, 2, 4, 6, and 8 hours post addition of EGM-2 to the top port of each device (scale bar = 250 μm). Yellow lines indicate the edge of the lumen. **(B)** Schematic of the analysis scheme employed to normalize the number of extravasated neutrophils. Neutrophils counted in Box 1 at all timepoints are divided by number of neutrophils counted in Box 2 at t=0 to normalized against the initial number of cells loaded into each lumen. **(C)** Normalized number of extravasated neutrophils in microfluidic devices with 2, 4, and 6 mg/mL collagen. Data quantified from 12 devices (2 mg/mL), 12 devices (4 mg/mL), and 12 devices (6 mg/mL) across 3 independent experiments and 3 neutrophil donors. All collagen concentrations were compared to each other at each time point. For each condition, estimated

marginal means and SEM were calculated and pairwise comparisons were performed with Tukey's adjustment. Error bars indicate the estimated marginal means ± SEM.

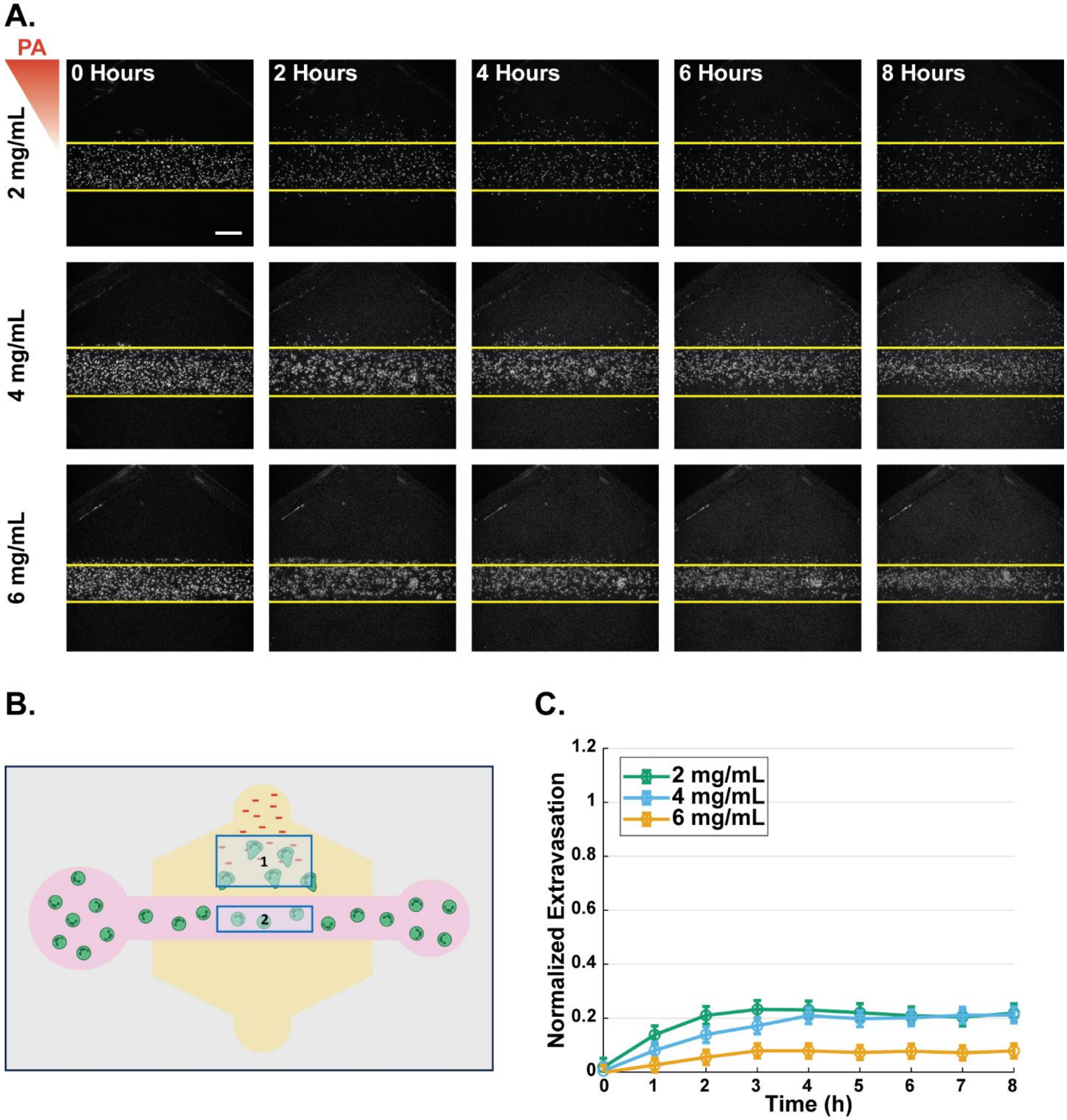

Supplementary Figure 4: Neutrophils do not differentially extravasate into varied collagen concentration matrices in the absence of an endothelium. (A) Representative images of extravasated neutrophils in 2, 4, and 6 mg/mL collagen at 0, 2, 4, 6, and 8 hours post stimulation with *P. aeruginosa* without an endothelium present (scale bar = 250 μm). Yellow lines indicate the edge of the lumen. The red triangle shows the initial bacterial gradient. **(B)** Schematic of the analysis scheme employed to normalize the number of extravasated neutrophils. Neutrophils counted in Box 1 at all timepoints are divided by number of neutrophils counted in Box 2 at t=0 to normalized against the initial number of cells loaded into each lumen. **(C)** Normalized number of extravasated neutrophils in microfluidic devices with 2, 4, and 6 mg/mL collagen. Data quantified from 15 devices (2 mg/mL), 20 devices (4 mg/mL), and 24 devices (6 mg/mL) across 6 independent experiments and 5 neutrophil donors. All collagen concentrations were compared to each other at each time point. For

each condition, estimated marginal means and SEM were calculated and pairwise comparisons were performed with Tukey's adjustment. Error bars indicate the estimated marginal means ± SEM.

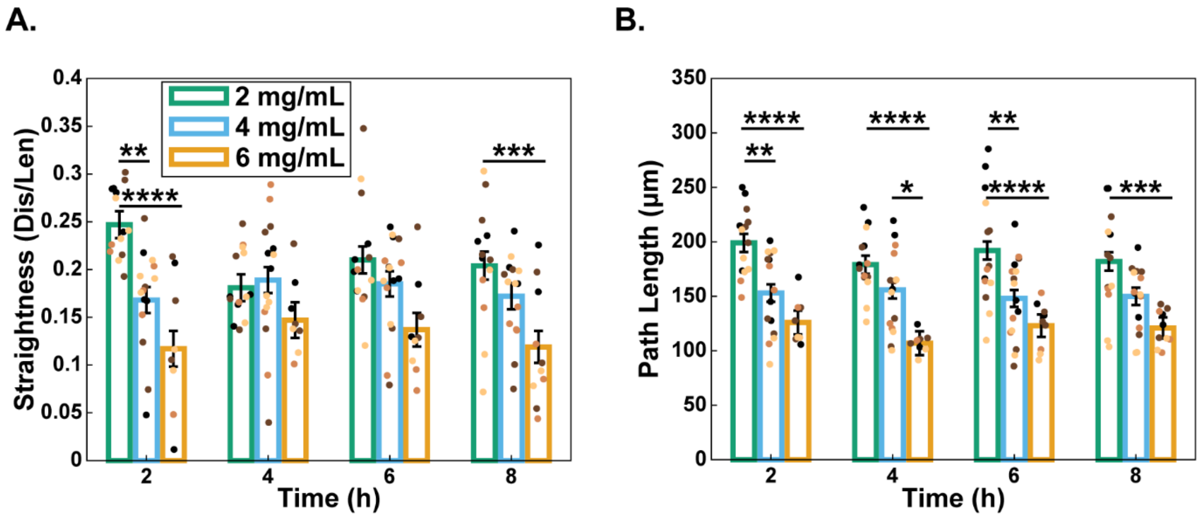

Supplementary Figure 5: Differential neutrophil migration length and straightness in various collagen ECMs post extravasation. Migration properties, **(A)** straightness and **(B)** path length, of neutrophils measured over 20-minute increments at 2, 4, 6, and 8 hours post stimulation with *P. aeruginosa*. Extravasated neutrophils, from 13 devices (2 mg/mL), 15 devices (4 mg/mL), and 10 devices (6 mg/mL) across 4 independent experiments and 4 neutrophil donors, were tracked using MTrackJ in Fiji. Dots indicate the average migration property for all neutrophils in each device and the dot colors indicate different independent experiments. All collagen concentrations were compared to each other at each time point. For each condition, estimated marginal means and SEM were calculated and pairwise comparisons were performed with Tukey's adjustment. Error bars indicate the estimated marginal means ± SEM. Asterisks indicate significance between conditions at a given timepoint (*p<0.05, **p<0.01, ***p<0.001, and ****p<0.0001).

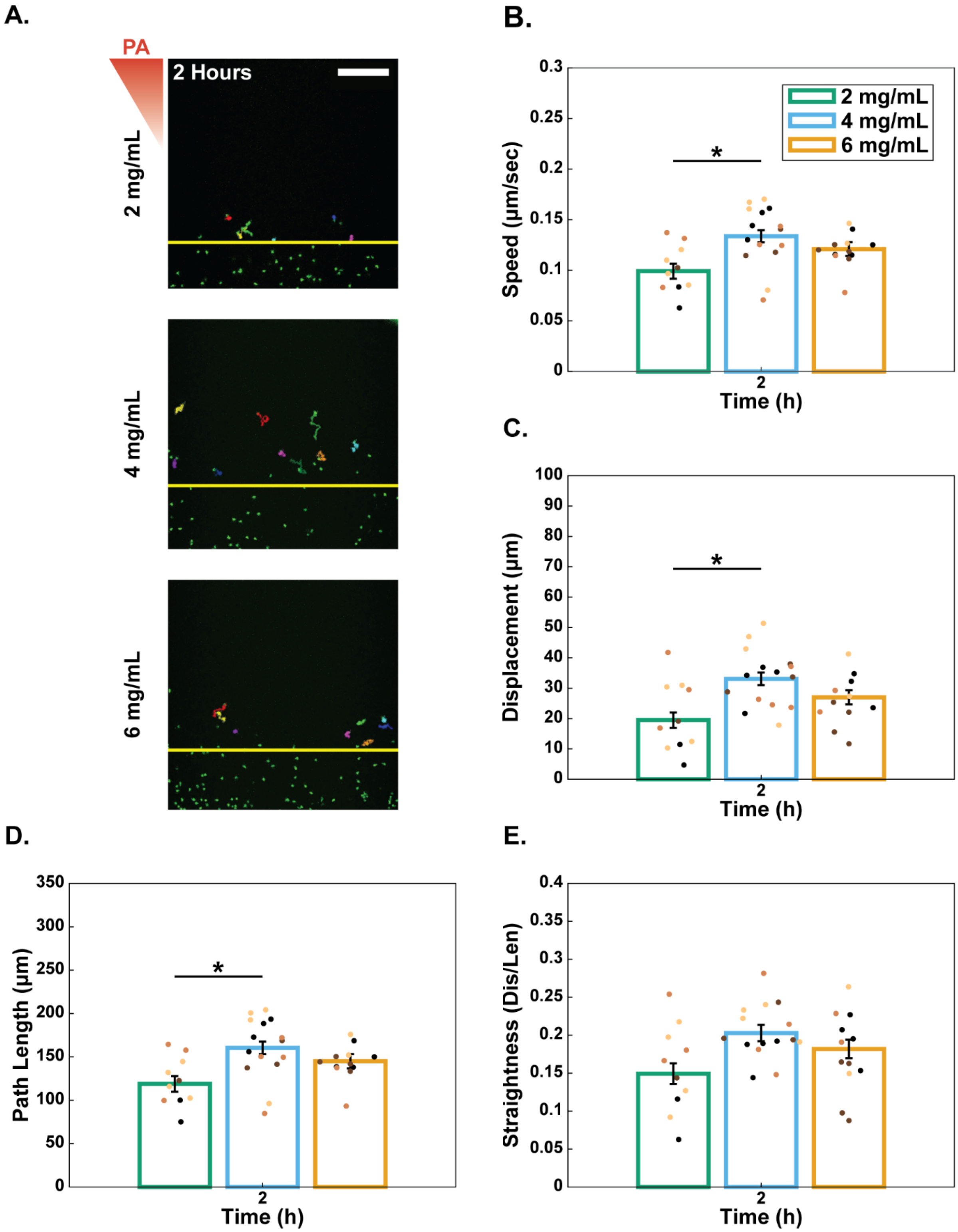

Supplementary Figure 6: Neutrophils migrate faster in 4 mg/mL collagen than 2 mg/mL collagen when the neutrophils do not extravasate through an endothelium. (A) Representative images and tracks of migrating neutrophils in 2, 4, and 6 mg/mL collagen 2 hours post stimulation with *P. aeruginosa* in the absence of an endothelium (scale bar = 250 μm). Each individual track is

shown in different colors. Yellow lines indicate the edge of the lumen. The red triangle shows the initial bacterial gradient. Migration properties, **(B)** speed, **(C)** displacement, **(D)** path length, and **(E)** straightness, of neutrophils measured over a 20-minute increment at 2 hours post stimulation with *P. aeruginosa*. Extravasated neutrophils, from 10 devices (2 mg/mL), 15 devices (4 mg/mL), and 13 devices (6 mg/mL) across 4 independent experiments and 4 neutrophil donors, were tracked using MTrackJ in Fiji. Dots indicate the average migration property for all the neutrophils in each device and the color of the dot indicates a given independent experiment. All collagen concentrations were compared to each other at each time point. For each condition, estimated marginal means and SEM were calculated and pairwise comparisons were performed with Tukey's adjustment. Error bars indicate the estimated marginal means ± SEM. Asterisks indicate significance between conditions at a given timepoint (*$p<0.05$).

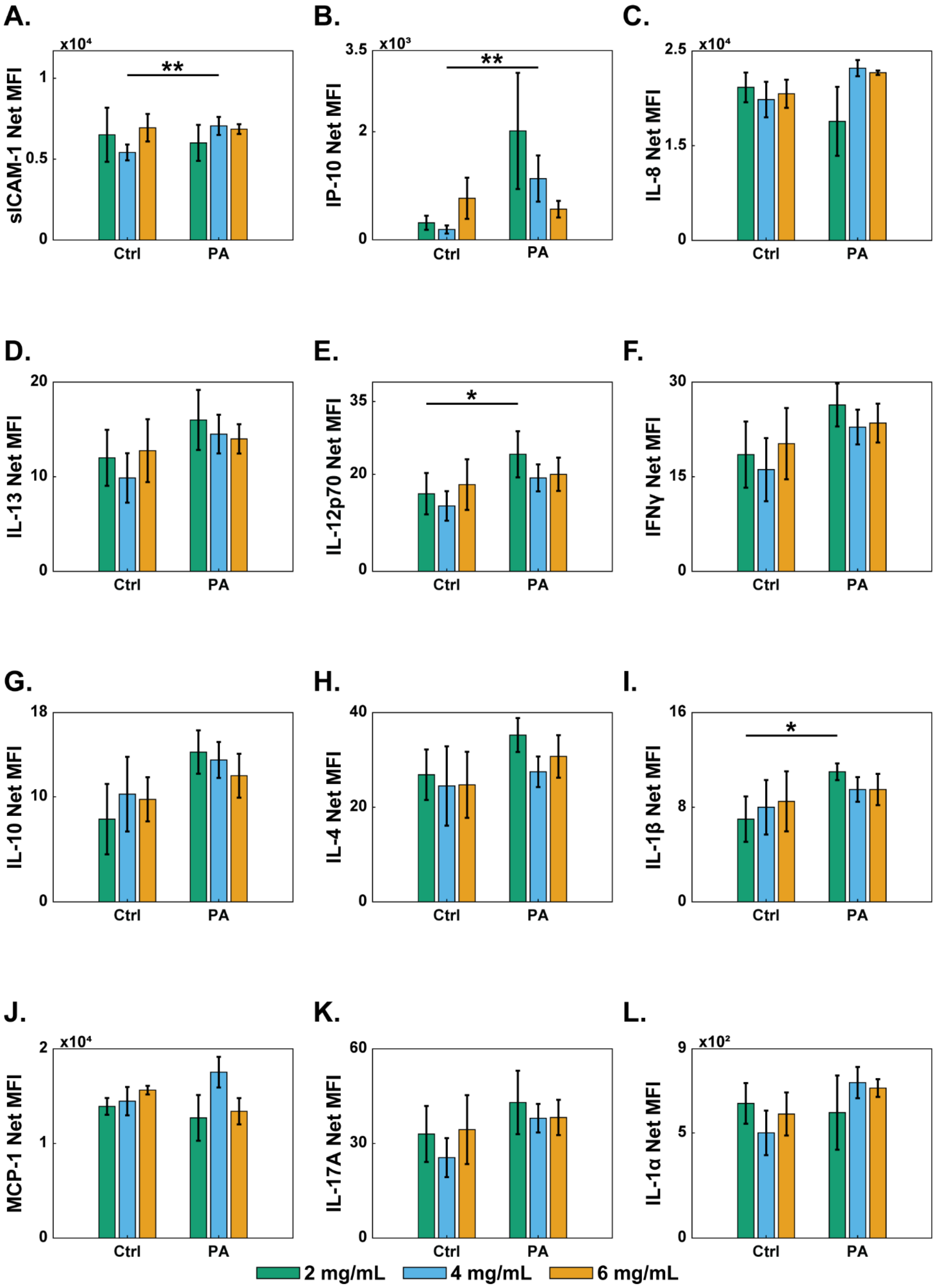
A.
x10⁴
sICAM-1 Net MFI
**
Ctrl
PA
B.
x10³
IP-10 Net MFI
**
Ctrl
PA
C.
x10⁴
IL-8 Net MFI
Ctrl
PA
D.
IL-13 Net MFI
Ctrl
PA
E.
IL-12p70 Net MFI
*
Ctrl
PA
F.
IFNγ Net MFI
Ctrl
PA
G.
IL-10 Net MFI
Ctrl
PA
H.
IL-4 Net MFI
Ctrl
PA
I.
IL-1β Net MFI
*
Ctrl
PA
J.
x10⁴
MCP-1 Net MFI
Ctrl
PA
K.
IL-17A Net MFI
Ctrl
PA
L.
x10²
IL-1α Net MFI
Ctrl
PA
2 mg/mL
4 mg/mL
6 mg/mL

Supplementary Figure 7: Multiplexed ELISA analysis of endothelial protein secretion profiles. Multiplexed ELISA of conditioned media from lumens of 2, 4, and 6 mg/mL collagen devices treated with EGM-2 (Ctrl) or *P. aeruginosa* in EGM-2 (PA) for 8 hours. Conditioned media was collected from 6 devices and pooled in 4 independent experiments. Samples were analyzed with a Luminex Magpix device and a ProcartaPlex Human inflammation Panel, 20Plex. Graphs show net mean fluorescence intensity (MFI) of soluble inflammatory markers: **(A)** sICAM-1, **(B)** IP-10, **(C)** IL-8, **(D)** IL-13, **(E)** IL-12p70, **(F)** IFNγ, **(G)** IL-10, **(H)** IL-4, **(I)** IL-1β, **(J)** MCP-1, **(K)** IL-17A, and **(L)** IL-1α. All collagen concentrations with and without *P. aeruginosa* were compared to each other for each inflammatory marker. For each condition, estimated marginal means and SEM were calculated and pairwise comparisons were performed with Tukey's adjustment. Error bars indicate estimated marginal means ± SEM. Asterisks indicate significance between conditions for a give cytokine (*$p<0.05$ and **$p<0.01$).
